# Communication practices for delivering health behaviour change conversations in primary care: a systematic review and thematic synthesis

**DOI:** 10.1186/s12875-019-0992-x

**Published:** 2019-08-03

**Authors:** C. Albury, A. Hall, A. Syed, S. Ziebland, E. Stokoe, N. Roberts, H. Webb, P. Aveyard

**Affiliations:** 1 Nuffield Department of Primary Care Health Sciences, University of Oxford, Radcliffe Observatory Quarter, Woodstock Road, Oxford, OX2 6GG UK; 2 Primary Healthcare Research Unit, Health Sciences Centre, Memorial University, 300 Prince Philip Drive, St. John’s, NL A1B 3V6 Canada; 3 Department of English Language, Faculty of Languages and Linguistics, University of Malaya, Kuala Lumpur, Malaysia; 4 School of Social Sciences, Brockington Building, Loughborough University, Loughborough, Leicestershire LE 11 3TU UK; 5 Bodleian Health Care Libraries, Knowledge Centre, ORC Research Building, Old Road Campus, Oxford, OX3 7DQ UK; 6 Department of Computer Science, Human Centred Computing (HCC) Group, University of Oxford, 39a St Giles, Oxford, OX1 3LW UK

**Keywords:** Primary care, Behaviour change, Health behaviours, General practice, Communication skills, Doctor-patient communication, Healthcare delivery

## Abstract

**Background:**

Clinical guidelines exhort clinicians to encourage patients to improve their health behaviours. However, most offer little support on how to have these conversations in practice. Clinicians fear that health behaviour change talk will create interactional difficulties and discomfort for both clinician and patient. This review aims to identify how healthcare professionals can best communicate with patients about health behaviour change (HBC).

**Methods:**

We included studies which used conversation analysis or discourse analysis to study recorded interactions between healthcare professionals and patients. We followed an aggregative thematic synthesis approach. This involved line-by-line coding of the results and discussion sections of included studies, and the inductive development and hierarchical grouping of descriptive themes. Top-level themes were organised to reflect their conversational positioning.

**Results:**

Of the 17,562 studies identified through systematic searching, ten papers were included. Analysis resulted in 10 top-level descriptive themes grouped into three domains: initiating; carrying out; and closing health behaviour change talk. Of three methods of initiation, two facilitated further discussion, and one was associated with outright resistance. Of two methods of conducting behaviour change talk, one was associated with only minimal patient responses. One way of closing was identified, and patients did not seem to respond to this positively. Results demonstrated a series of specific conversational practices which clinicians use when talking about HBC, and how patients respond to these. Our results largely complemented clinical guidelines, providing further detail on how they can best be delivered in practice. However, one recommended practice - linking a patient’s health concerns and their health behaviours - was shown to receive variable responses and to often generate resistance displays.

**Conclusions:**

Health behaviour change talk is smoothly initiated, conducted, and terminated by clinicians and this rarely causes interactional difficulty. However, initiating conversations by linking a person’s current health concern with their health behaviour can lead to resistance to advice, while other strategies such as capitalising on patient initiated discussions, or collaborating through question-answer sequences, may be well received.

**Electronic supplementary material:**

The online version of this article (10.1186/s12875-019-0992-x) contains supplementary material, which is available to authorized users.

## Background

Health behaviours such as excessive alcohol consumption, lack of physical activity, and smoking are a major cause of morbidity and chronic disease. Clinical guidelines exhort clinicians to encourage patients to improve their health behaviours in order to reduce the incidence of associated diseases [1–5]. Whilst these guidelines provide detailed advice on treatment options, most offer little support on how to have these conversations in practice. NICE guidelines on weight management, for example, state that clinicians should “Raise the issue of weight loss in a respectful and non-judgmental way” [4] but do not detail how this is best achieved.

Clinicians have reported reluctance to talk about health behaviours with patients, and oriented to a lack of support from guidelines. They report a number of barriers, including concern that talking about health behaviours could cause offence [6, 7], and a lack of knowledge about how to carry out these conversations in ways which are likely to be well received. Clinicians want more support regarding how to talk about health behaviour change with patients [7].

Patients have also reported issues discussing their health behaviours with their physicians in consultations. For example, patients have found particular ways their clinician discussed health behaviours created negative feelings [8, 9]. These studies often used post-consultation interviews with patients to explore their perceptions and experiences of the conversations they had with the clinician during the consultation, they do not analyse the conversations that were actually carried out. Consequently, there are no specific data on the precise type of talk that led to these feelings.

The fields of conversation analysis and discourse analysis offer relevant research which can address this gap. What we currently know about this aspect of care is derived from after-the-fact reports from patients or clinicians [6, 10], which can be subject to recall or social desirability biases [11]. However, several studies have used more objective methods, exploring consultation recordings. It is now timely for us to synthesise the evidence in this area and, where possible, make recommendations for clinical practice. Conversation and discourse analyses systematically explore recorded consultations allowing empirical observation of how clinicians can successfully negotiate complex conversations and facilitate development of specific recommendations for practice. Conversation analysis involves analysing sequences of interaction [12]. This method looks at what is said, how it is said (including speed, pitch, pauses, and body movement) and what happens next [13]. Researchers examine large numbers of similar types of conversations, for example treatment recommendations, or requests, and identify common patterns in the interactional sequence [14]. This detailed micro-level analysis of interaction enables researchers to understand how communication practices function in everyday life, and which patterns of communication are likely to produce certain responses from conversational partners. These methods allow researchers to qualitatively identify “the techniques and competencies involved in successful and unsuccessful conversation” [15] at a level of detail which cannot be captured through coding frameworks, interviews, or theoretically interpreted studies. These observational methods have been used to inform the training of healthcare professionals to deliver interventions [16], to make practice and policy recommendations [13] and to inform clinical guidelines [17, 18]. These observational methods have been used to inform the training of healthcare professionals to deliver interventions [16], to make practice and policy recommendations [13] and to inform clinical guidelines [17, 18].

This review explores health behaviour change talk (HBCT) used by clinicians when communicating with patients in a healthcare setting. We define ‘health behaviour change talk’ as talk designed to change health behaviours. Activities classified as ‘health behaviours’ will be patterns of lifestyle associated behaviour which might impact on patient health (further definitions are provided in Table 1). We aim to identify and synthesise evidence from conversation and discourse analytic studies regarding how clinicians communicate with their patients about health behaviour change (HBC), and the responses each practice is likely to generate from patients. We also aim to establish gaps in current evidence, and highlight recommendations for practice, exploring how results from this review articulate with current clinical guidelines.

**Table 1:** Key terms

Health behaviours - patterns of lifestyle associated behaviour which might impact on patient health	
Health Behaviour Change talk – turns at talk designed to change health behaviours. ‘Talk’ comprises aspects of interaction which includes both what is said, but also how it is said. This incorporates aspects of word choice, grammar, conversational action, pitch, pace , intonation, and embodied conduct.	
Resistance displays– Interactionally dispreferred responses which may be delayed and mitigated, and which stall the progressivity of the conversational sequence. Resistance can range from no response, a minimal response, or not displaying alignment to the course of action initiated in the prior turn; e.g., behaviour change. Resistance occurs moment-by-moment through an interaction, and is managed by participants during the interaction [19, 20].	

## Methods

We aim to synthesise evidence from conversation and discourse analytic studies of recorded healthcare interactions. Approaches to data analysis in conversation and discourse analysis differ from more conventional qualitative methods. Therefore, we followed established recommendations for reviewing, quality appraising, and synthesising this type of data [21], and our reporting follows ENTREQ guidelines.

### Inclusion and exclusion criteria

Studies which met the following criteria were included: naturally occurring talk in interaction; audio or audio-visually recorded interactions; healthcare professional/patient interactions; interactions occurring within a healthcare setting; conversation or discourse analytic methodology; peer-reviewed papers or published book chapters, and behaviour intended to reduce long-term health risk because the behaviour is sustained or repeated over the long-term; e.g., stopping smoking or safer sex practices.

We excluded studies which solely used coding frameworks; group interactions; interpreter mediated encounters; encounters that have been translated into English; dissertations; book reviews; conference proceedings, and interactions including proxy decision making. No other exclusions have been placed on the disease, condition, or healthcare domain being studied. No limits were placed on healthcare professionals’ roles, patients’ reasons for visit, or any patient characteristic.

Screening was conducted using Covidence systematic review management software. All titles were screened by a single reviewer (CA), and those which did not meet the inclusion criteria were excluded at this stage. Next, abstracts of remaining titles were screened for eligibility independently by two reviewers (CA and AH, PA, or AS), and conflicts were resolved through discussion, or involvement of third team member (SZ or PA). Full-texts were also independently screened for inclusion by two reviewers (CA and AH, PA, or AS). Our protocol was registered with Prospero: International Prospective Register of Systematic Reviews and is available online. Prospero Protocol ID 42016041782.

### Data sources

We searched the following databases from database inception to March 2018: MEDLINE (OvidSP)[1946-present]; Embase (OvidSP)[1974-present]; Web of Science Core Collection (Thomson Reuters)[1945-present]; AMED (OvidSP)[1985-present]; CINAHL (EBSCOHost)[1982-present]; PsycINFO (OvidSP)[1967-present]; Scopus; Sociological Abstracts (CSA) [1952-present]. We did not limit by date because conversation analysis emerged as a discipline in 1960s, and discourse analysis in the 1950s. Restrictions were applied to specify human subjects and English language. We used two different strategies to capture the variety of reporting in this field. The first search strategy was designed to identify relevant literature which focussed on a specific health behaviour (such as “weight loss”, or “smoking cessation”) – this strategy used free-text terms using the databases’ default keyword search. The second was designed to identify literature which may focus on a behaviourally-related action (such as “adherence” or “motivation”), rather than specific behaviour – this strategy used a combination of free-text terms using the databases’ default keyword search along with database specific subject headings where available. In addition, we screened bibliographies of included full-texts; specialist online discussion lists; and review team knowledge and contacts. All searches were conducted from January to March 2016. Searches were updated in March 2018. The research strategy was designed with advice from an information specialist (NR). The full search strategy is available in Additional file 1.

### Data extraction

Data extraction was conducted independently by both CA and AH. Data extraction materials used by Parry et al. [13] were adapted to facilitate extraction of the types of health behaviour discussed, healthcare setting, and implications for practice. Information was extracted regarding study characteristics; the types of talk used by clinicians when discussing HBC; and, where possible, the responses these received from patients.

### Quality appraisal and synthesis

We followed existing practices for appraising the quality of studies which use conversation or discourse analysis [21]. The unique features of conversation and discourse analysis, where interactional practices and their consequences are identified and described, mean that traditional methods of quality assessment are not possible. Following Parry and Land [21], we identified the type of data analysis; how many examples were collected; and the depth of analysis used in each study. This appraisal showed that some studies conducted a detailed sequential analysis of a number of similar interactions and offered comprehensive results on conversational practices and their relationship to patients’ responses. Others explored conversations in less depth, but nevertheless provided evidence on the presence or absence of particular conversational practices. All studies were included in data synthesis.

Synthesis followed an aggregative thematic synthesis approach [22]. This involved line-by-line coding of the results and discussion sections of included studies, and the inductive development of descriptive themes. Similar themes across studies were then grouped hierarchically using the one sheet of paper (OSOP) technique [23], where conversational practices were summarised to produce top-level descriptive themes. This aggregative approach is in line with current practice for synthesising conversation and discourse analytic studies [21]. It ensures results are ‘accumulated’ and ‘summarised’, rather than ‘transformed’ [24]. This approach allowed reporting to closely reflect the conversational practices demonstrated in included studies and did not seek to generate new theoretical concepts. Synthesis was conducted by one reviewer (CA) with a second (SZ) providing input on final grouping of descriptive themes. Data were coded and managed using NVivo 11 for Mac.

## Results

### Included studies

**Fig. 1:**
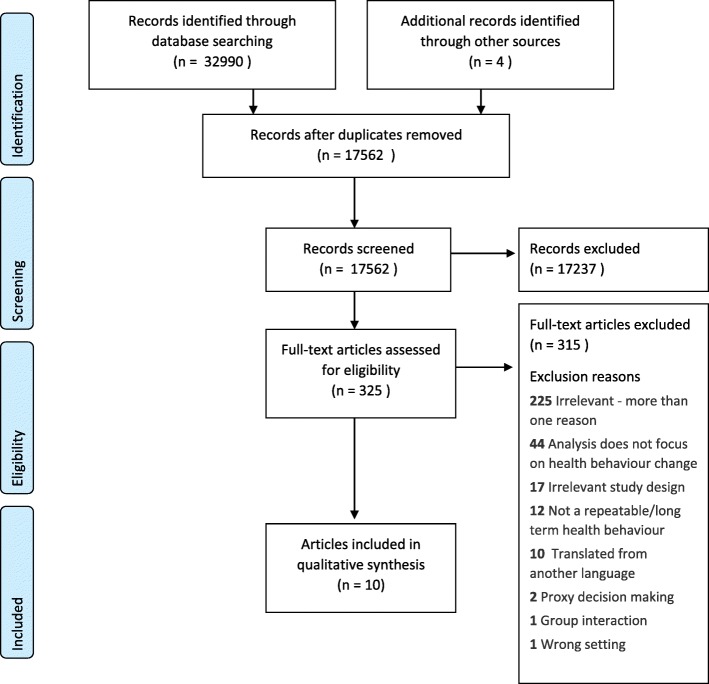
Prisma Flow Diagram

Of the 17562 studies identified through systematic searching, ten papers from eight unique observational studies fulfilled the inclusion criteria. Figure 1 illustrates the screening and assessment process. Included studies were conducted in four countries (USA; Canada; Australia; and UK) and in two healthcare settings (primary care, and sexual health clinics). Studies were published between 1992 and 2014. The characteristics of included studies are described in Table 2.

**Table 2:** Description of included studies

Authors	Country	Health Behaviour (s)				Participants	Setting	Method	Audio/ video	Corpus size	Recordings used for analysis
		Weight management	Smoking cessation	Promoting lower alcohol consumption	Safer sex advice						
Cohen, D.J. et al. 2011 [25]	USA	✓	✓			General practitioner / patient	General Practice	Conversation analysis	Audio	811	541
Collins, S. et al. 2005 [26]	UK	✓				General practitioner / patient	General Practice	Conversation analysis	Audio	168	80
Freeman, S.H. 1987 [27]	USA	✓	✓	✓		General practitioner /patient & nurse/patient	General Practice	Conversation analysis and observational methods	Video	200	200
Kinnell, A. & Maynard, D. 1996 [28]	USA				✓	Counsellor / patient	Primary Care	Conversation analysis & ethnography	Audio	66	25
Pilnick, A. & Coleman, T. 2003 [29];2010 [30]	UK		✓			General practitioner / patient	General Practice	Informed by conversation analytic principles	Video	538	47
Silverman, D. et al. 1992a [31]; 1992b [32]	USA UK				✓	Counsellor/ patient	Primary Care	Conversation analysis	Audio	100	100
Tapsell, L. 1997 [33]	Australia	✓				Dietitian / patient	General Practice	Conversation analysis	Audio	30	30
Thille, P. et al. 2014 [34]	Canada	✓				Family health team member /patient	General Practice	Discourse analysis	Audio	12	12

From the eight unique studies included, seven papers, from six studies, were from general practice [25–27, 29, 30, 33, 34] and three papers, from two studies, were from primary care [28, 31, 32]. Some papers reported multiple health behaviours, or analysed HBCT from more than one healthcare professional. The behaviours discussed were weight management (5 studies); smoking cessation (3 studies); safer sex practices (2 studies); and lowering alcohol consumption (1 study). Healthcare professionals engaging in HBCT were general practitioners (4 studies); sexual health counsellors (2 studies); dieticians (1 study); nurses (1 study); and family health team members (1 study).

All studies conducted a sequential analysis of recorded talk. Seven of the eight used a conversation analytic methodology [25–33] and one used discourse analysis [34]. Seven were also multi-case analyses [25–33], while one was a single-case study [34].

Most studies focused on clinician communication behaviours. Only one study focused in detail on patient responses to HBCT [29]; five studies outlined, to varying degrees, typical patient responses to the HBCT which was presented without these analyses being the main focus of the paper [25–28, 30–32]; and one explored HBC conversations between one patient and two different healthcare professionals [34]. HBCT which produced patient resistance displays (see Table 1) were highlighted by all papers, and in all instances, patient response was used as a measure for the efficacy of HBCT. All studies used audio data, and two used audio-visual data [27, 30].

### Aggregative thematic synthesis

Included studies were coded and thematically aggregated. Initial coding produced 102 codes across all 10 included studies, resulting in a total of 14 top-level descriptive themes [24]. Conversational practices which were only described in one study are not reported here. Therefore, we present seven top level themes. To optimise the clinical relevance of the conversational strategies used by clinicians, these themes are presented separately for each stage of the behaviour change discussion [21]. The stages include initiating HBCT; carrying out HBCT, and closing the HBCT. Quotations are presented to illustrate conversational practices, and transcriptions have been adapted to verbatim from the original studies. A description of the frequency of each conversational practice, across studies, is presented in Table 3. Table 4 shows each conversational practice and the response it is likely to receive from patients.

**Table 3:**
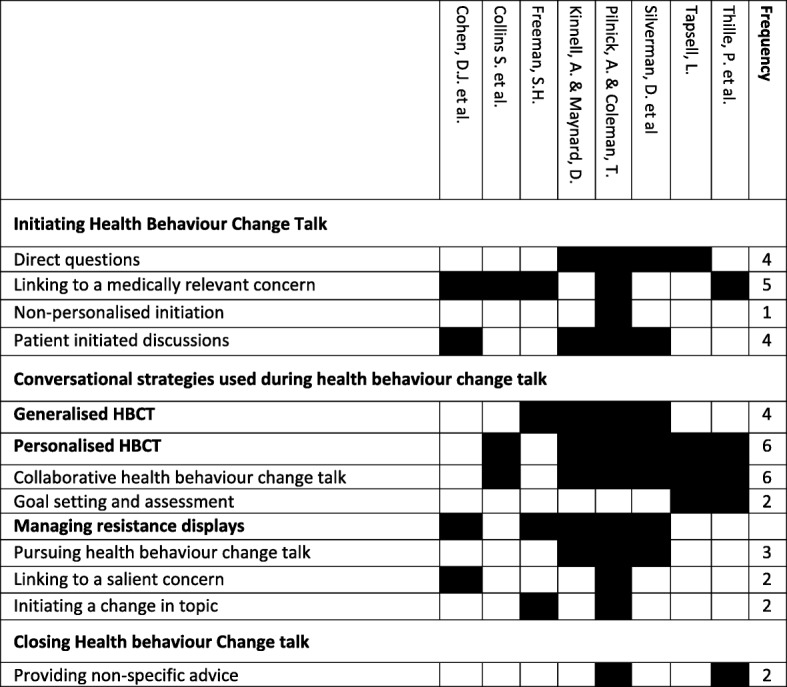
Frequency of conversational practices across included studies

**Table 4:** Description of conversational practices used

Type of HBCT	Description of HBCT	Patient response	Recommend strategy
Conversational strategies used for initiating health behaviour change talk			
1. Direct questions	Health behaviours are raised as a direct question, targeting a specific health behaviour, such as ‘do you smoke?’	Undesirability of health behaviour may be acknowledged	?
2. Linking to a medically relevant concern	Health behaviours are linked with an associated, medically relevant, concern	Varying efficacy. Potential for strong resistance	X
3. Patient initiated discussions	Health behaviour change discussions are initiated by a patient	Receptive to subsequent health behaviour change talk	✓
Conversational strategies used during health behaviour change talk			
1. Generalised HBCT	Not tailored to specific patients’ concerns or conditions. HBCT is framed as relevant for ‘patients in general’.	Avoids potential for resistance but does not implicate patients to engage in future action.	?
2. Personalised HBCT	HBCT was tailored to individual patient, and often involved patients in decision making and elicited their views	Facilitates patient engagement. Can be perceived as intrusive. Potential to implicate patient action.	
a. Collaborative HBCT	Inviting and accommodating a patient’s perspective and presenting decisions as the patient’s choice	Displays of uptake	✓
b. Goal setting and assessment	HBC goals are set and reviewed	Potential for resistance if biomedical outcomes, rather than changed behaviours, are prioritised.	✓
3. Managing resistance to behaviour change talk	Addressing or avoiding patient resistance displays.	Patient response depends on strategy used (below)	
a. Pursuing health behaviour change talk	Continuing with HBCT despite patient resistance displays.	Patient response depends on strategy used.	?
b. Initiating a change in topic	Clinicians avoid addressing displayed resistance, and change the topic	Unlikely to result in further resistance	✓
Conversational strategies used for closing health behaviour change talk			
1. Non-specific Advice	HBCT is vague, non-personalised, and lacks a next action step	No overt resistance, but no evidence for effectiveness in facilitating behaviour change	X

#### Initiating HBCT

All studies included in this review documented strategies which are used by clinicians to initiate these conversations. These strategies were: direct questions [28, 29, 32, 33]; linking HBC to a medically relevant concern [25–27, 29, 34]; and patient initiated discussions [25, 28, 30, 31] . The following sections will discuss each of these in detail. One paper used patient responses as a unit of analysis [29], while others used them as proxy measures for the success or failure of clinicians’ talk.

##### Direct questions

Health behaviours can be raised as a direct question targeting a specific health behaviour, such as ‘*do you smoke?*’ [29], or “*When the two of you engage in any type of sexual activity do you use safe sex?*” [28] . Four studies; two from sexual health clinics [28, 32], and two from primary care [29, 33] reported this practice. One primary care study [29] documented direct questions as the most common way of initiating HBC discussions about smoking cessation.

Only one study, from primary care, described patient responses to these direct questions [29]. It outlined a pattern where patients acknowledged the undesirability of their behaviour and provided more information, such as recounting attempts to change behaviours, or giving rationales for not doing so. Clinicians then used information provided by patients to inform subsequent discussion [28, 32, 33].

##### Linking to a medically relevant concern

There are opportunities to initiate HBCT when an associated, medically relevant, concern is discussed. Five studies from primary care [25–27, 29, 34] reported that linking health concerns and health behaviours was commonly used to initiate HBCT, and three of these explored this phenomenon in detail [25, 27, 29]. Articulating a link between an existing health concern and a health behaviour may be expected to facilitate HBCT by emphasising its personal relevance for a particular patient. However, this strategy did not always achieve this.

Two primary care papers [25, 29] found that this method was unsuccessful when the link was made to a health concern which was not salient for the patient. For example, in one study a clinician explained weight loss would be beneficial, but the patient resisted this advice [25]. However, when the same clinician linked dieting, weight loss, and reduced risk of mortality (associated with the patient’s status a new parent), the patient engaged with and oriented to this as salient (Excerpt 1):

*Doctor: Okay. Alright. We want to – you know keep you around as long as possible*

*Patient: Yes*

*Doctor: since …you’ve got a little one. So.*

*Patient: Yeah.*

*Doctor: I would recommend exercising and really watching your sugars.*

*Excerpt 1 Cohen et al. 2011*

Evidence from one paper showed that links to salient concerns were also rejected [25]. In Excerpt 2, following the clinician’s link between their smoking and respiratory infection, this patient responds in a louder voice overtly resisting the association, and saying instead it was air conditioners on the bus which caused cold symptoms:

*Doctor: You still smoking?*

*Patient: “((The patient’s voice is much louder during this turn)) That’s from getting off- ((audible exhale)) actually being on the bus and they had the air conditioners up up and don’t turn them down. I caught a cold from there.”*

*Excerpt 2 Cohen et al. 2011*

Pilnick and Coleman state that these displays of resistance in response to linking are ‘rarely seen in other medical consultations’ [29]. Conversely Freeman, states that linking HBC with a well-known illness condition was the most frequent and ‘least disruptive’ pattern which was observed in her US primary care study [27].

Two papers [25, 29] use the data to infer that there are strong moral implications of associating a patient’s illness with their behaviours. In doing so this evokes connotations that the patient is responsible and can be blamed for their own illness. Patients appeared to be perceiving that clinicians were undermining the legitimacy of a patient’s illness and their request for medical assistance. These moral elements may result in the significant displays of resistance seen by Cohen et al, and Pilnick and Coleman in response to linking health behaviours and medically relevant concerns.

Rather than linking to *initiate* HBCT, Pilnick and Coleman [29] argue that a general, non-personalised entry into HBCT (e.g. Establishing smoking is a problem), securing agreement on this statement from the patient, and then moving to a more personalised discussion, would be less likely to generate resistance.

##### Patient initiated discussions

HBC discussions were sometimes initiated by a patient, rather than a clinician. Four studies; two from primary care [25, 29] and two from sexual health clinics [28, 31], examined HBCT in this context. Patient initiated HBCT was reported to be rarer than clinician initiated HBCT [29, 31]. Patients were shown to have initiated HBCT either through asking directly for HBC advice, or raising a potentially relevant topic which provided the clinician with an opportunity to move forwards with HBCT (see linking above). The authors hypothesised that, through raising the topic of health behaviours themselves, patients were indicating that they were receptive to behaviour change advice [28].

#### Conversational strategies used during HBCT

Studies in this review showed that clinicians used two clear strategies for delivering HBCT these were ‘generalised HBCT’ (four studies) and ‘personalised HBCT’ (six studies). Additionally, five studies outlined strategies that clinicians used to manage patient resistance during HBCT [25, 27–29, 31]. These strategies, and the responses they were likely to receive are explored below.

##### Generalised HBCT

HBCT was sometimes delivered in ways which can be seen to be true for ‘patients in general’ rather than tailored to a specific person. Four studies; two from primary care [26, 29], and two from specialised sexual health clinics [28, 32], explored how generalised HBCT was given, and the responses these produced from patients. These studies showed that HBCT can be generalised through avoiding tailoring to a specific patient by talking hypothetically [28, 29], or delivering ‘information’ rather than ‘advice’ [26, 29, 32]. This is exemplified in Excerpt 3:

*Counselor: .hhhh Now when someone er is tested and they have a negative test result .hh it’s obviously dealuhm that they then look after themselves to prevent any further risk of*

*Patient: Mm hm*

*Counselor: infection. .hhhh I mean obviously this is only possible up to a point because if .hhh you get into a sort of serious relationship with someone that’s long term .hh you can’t obviously continue to use condoms forever, .hh Uhm and a point has to come where you make a sort of decision uhm if you are settling down about families and things that you know you’d- not to continue safer sex*.

*(15 lines omitted)*

*Now whe- when someone gets a positive test result er: then obviously they’re going to ke- think very carefully about things, .hhhh Being HIV positive doesn’t necessarily mean that that person is going to develop aids later on*.

*Excerpt 3 Silverman et al., 1992*

A non-personalised approach was presented as a way to acknowledge the delicacy of HBC discussions. In general this non-personalised format was reported to produce acceptance [26] or minimal acknowledgment from patients. Two studies stated that this talk was largely clinician led [26, 32]. All studies showed that this type of talk mitigated the risk of confrontation, as the health behaviours discussed were not overtly presented as those undertaken by that particular patient. One study concluded that non-personalised HBCT was shorter than personalisation, fitting better with the time constraints of healthcare consultations [32]. However, two studies stated that non-personalised HBCT could also be problematic as, although patients rarely resist, they may not have heard advice as relevant for them [29], or may have rejected HBC [28]. Based on the minimal patient responses this practice often received, one study hypothesised that untailored, unilaterally delivered information may not be adequate in motivating behaviour change [32].

##### Personalised HBCT

The practice of tailoring and personalising HBCT for a specific patient, rather than for ‘patients in general’, was observed in six studies in this review; four from primary care [26, 29, 33, 34] and two from sexual health clinics [28, 32]. This personalised HCBT consisted of two distinct communication practices. These two practices, and their associated patient responses, are outlined below.

*Collaborative HBCT*

Four studies from primary care [26, 29, 33, 34] and two studies from sexual health clinics [28, 32] examined how HBCT was built collaboratively. This was done through inviting a patient’s perspective and accommodating this throughout HBCT by tailoring responses in line with their perspectives (Excerpt 4), or acknowledging HBC, or the degree of HBC, as the patient’s choice (Excerpt 5):

*Clinician: Lite White milk. Have you tried another type of milk?*

*Patient: Shape and skim milk*

*Clinician: What do you think of Shape?*

*Patient: Shape’s not bad. I don’t like the skim milk except the one you buy on the shelf, that’s nice.*

*Clinician: Yeah. um um so would you be happy changing to Shape d’ye think?*

*Patient: yeah, it wouldn’t worry me. It’s pretty much the same as Lite White only a little bit less*

*Clinician: yeah, t’ it does have less fat um and that would, that would contribute considerably if you used uh Shape all the time. Do you have any problems with that?*

*Patient: No not at all.*

*Excerpt 4 Tapsell, 1997*

*Clinician: And is it 2 days a week, is that what you think you can maintain, or maybe once a week? Or what would be best? .*

*Excerpt 5 Thille et al., 2014*

There was evidence that this was used by clinicians to inform joint decision-making in a consultation. Such sequences usually led to clinicians inviting patients directly to comment on and agree with proposed HB changes that emerged from this joint enterprise, and patients responded with uptake displays. However, Pilnick and Coleman found that, if the patient’s opinion was sought and HBCT initiated immediately, without asking further questions and tailoring advice, less uptake, or resistance occurred [29]. Additionally, one study reported that collaborating using a question/answer pattern appeared intrusive [28], although the evidence presented was sparse; and another that it and took longer than other methods [32]. However, although this approach had potential for variability, collaborative HBCT was reported to most often result in displays of uptake from patients, rather than resistance, which likely indicate receptivity to HBC.

*Goal setting and assessment*

Two studies, one from primary care [34] and one from a sexual health clinic [32], documented goal setting and assessment as components of HBCT. Some goals were clinically oriented, and set or assessed with comparison to guidelines or biomedical recommendations; whilst others were related to self-improvement, or comparison with others [32, 34]. There was no evidence on patients’ responses to these goal-setting strategies and no data on which circumstances they could be best used.

Thille et al. [34] found that, during goal assessment in a primary care weight loss review, there was potential for disruption if only the desired outcome (e.g. weight loss) was celebrated and emphasised rather than the HBC itself (making dietary changes). The evidence is limited as it is generated from one single case analysis. However, the authors concluded that emphasising personal responsibility for clinical outcomes generated resistance displays.

##### Managing resistance displays

Five studies, three from primary care [25, 27, 29], and two from sexual health clinics [28, 32] explored how clinicians responded to resistance displays. Resistance displays were sometimes minimal responses, no responses, proposition of alternative views, or overt patient rejection of HBCT. Two broad strategies emerged where doctors dealt with resistance displays by either initiating a change in topic, or continuing to pursue HBCT.

*Pursuing HBCT*

Three of the five studies explored how clinicians pursued HBCT when faced with patient resistance displays [28, 29, 32]. Most studies showed that pursuing HBCT following resistance escalated resistance displays. However evidence from two studies showed that if resistance occurred following a link between weight and health, pursuing talk by ‘linking to a salient concern’ [25, 29] often addressed resistance to the initial link, and allowed for more productive HBCT [25, 29].

*Initiating a change in topic*

Two studies from primary care [27, 29] examined what happened when clinicians changed topic in response to resistance displays. Rather than pursuing HBCT, clinicians in these cases avoided addressing displayed resistance, and changed topic to discuss less-delicate matters. This is illustrated in Excerpt 6 where the patient displays resistance to discussion of smoking and the doctor responds by changing the topic to talk about medication. Both studies which examined this topic demonstrated that following this strategy enabled HBCT to be discontinued successfully and the normal business of a consultation resumed with minimal disruption.

*Doctor: you smoke?*

*Patient: yes*

*Doctor: there’s some things you can do these days that really help with cutting down… with quitting.*. *cause that is really something you should think about*

*Patient: [5 sec silence]*

*Doctor: well.*. *. so*. *.*. *how’re you getting along with the Tagamet so far? seem okay?*

*Patient: seems okay.*. *. no problem*

*Doctor: no problem.*. *. good.*

*Excerpt 6 Freeman, 1987*

#### Closing HBC discussions

We identified a lack of evidence on closing health behaviour change talk. Only two studies from primary care discussed methods for closing HBC discussions [30, 34], and both oriented to difficulties in doing so effectively.

##### Non-specific advice

These two studies showed how, when closing HBCT, clinicians often presented the harms of a health behaviour, with no specific follow-up advice. One paper additionally found that clinicians did not assess a patient’s capability to carry out behaviour change [34] and a second demonstrated that they also gave vague non-expert advice [30]. We have termed this approach ‘non-specific advice’ as the HBCT was vague, non-personalised, and lacked a next action step:

*Doctor: The best way is just to think about it, think about how you’d stop and when you’d stop rather than just having it as something in the future.*

*((Patient doesn’t respond to this utterance, and gets up ready to leave)).*

*Excerpt 7 Pilnick & Coleman, 2010*

This non-specific advice does not acknowledge a patient’s health behaviour as a medical problem nor does it give specific instructions to facilitate change. Pilnick and Coleman’s study found that this technique expedited closing and did not overtly generate resistance. However, Pilnick and Coleman [30] state that patients oriented to a ‘to a lack of success’ in providing a HBC solution, and hypothesised that this may be associated with a lack of action to change health behaviours.

## Discussion

### Summary of key findings

In ten papers from eight studies, we found that practitioners used a range of strategies to talk about HBC. We grouped these into seven categories, and three domains which indicated their positioning within a consultation. These domains are initiating health behaviour change; carrying out HBCT, and closing HBCT.

HBCT was shown to be initiated through ‘direct questions’; ‘linking to a medically relevant concern’; and ‘capitalising on patient-initiated discussions’. There was strong evidence that patient-initiated talk was successful in terms of patient receptivity to HBCT, while HCP linking of health behaviours with health conditions was shown to be a delicate strategy which could generate resistance displays from patients. Two methods were identified for delivering HBCT, once initiated. These were ‘generalised’ and ‘personalised’ HBCT, and there were several ways to implement each of these. ‘Generalised HBCT’ was not overtly presented as personally relevant for patients. Evidence indicated that presenting health behaviour change as ‘information, for people in general’, avoids potential resistance displays. ‘Personalised HBCT’ was tailored for specific patients. It was reported to be well received in general. However, there was some limited evidence that a shared understanding of the relevance of HBC was required before being personalised.

We identified two strategies for managing resistance displays; either ‘pursuing HBCT’, or ‘dropping the topic’. In general, pursuit escalated resistance displays, whilst dropping the topic allowed normal business to be successfully resumed. One potentially useful method of pursuit was to link to a salient concern. This showed that, whilst linking may be a risky way to *initiate* health behaviour change talk, it may be a helpful way to address resistance displays if the concern is salient for patients. We identified a clear dearth of evidence on closing HBCT. Only one practice was identified, which was provision of ‘non-specific’ advice. This was reported to expedite closings, but was shown to be vague, and the authors hypothesised that the minimal responses that were received, a lack of providing an affirmative next step meant that it was unlikely to motivate behaviour change.

### Strengths and limitations

The key strength of this review is the application of systematic review methods to a field to which such methods have been rarely applied. Doing so allowed us to provide the most comprehensive assessment of the evidence on this key public health priority that clinicians struggle with because finding the words is a challenge. We used a systematic search strategy, but many studies were published in social science journals and some of these do not use MeSH terms so it is possible that we have missed relevant studies. We supplemented this with a comprehensive search strategy with a good deal of full text screening and forward and backward citation checking, and consulted experts, suggesting we have identified the key studies. The methods we used were appropriate to capture key studies; identify and aggregate conversational practices across studies, and foreground their clinical relevance.

On the other hand, the review had limitations. The chief of these is that we used patient response as a proxy for conversational effectiveness. None of the studies reviewed collected subsequent data on future behaviour change and/or whether the likelihood of change depended upon the preceding consultation. Only one of the included studies used video data, so we were unable to review the role of embodied communication. Another limitation of this review was that the review comprised only ten papers from eight studies. The data available are unlikely to comprise a complete overview of all interactional practices used by clinicians when delivering HBCT, and most of the included studies were from general practice. Furthermore, it is possible that certain conversational practices may be more or less appropriate for different health behaviours or different healthcare settings, but due to a dearth of current literature these could not be identified. These available data highlight that more research is needed to examine how health behaviour change talk is carried out in practice. Some older studies explored clinical circumstances which may now have changed. However, there is evidence that communication practices are relatively consistent [35, 36]. This is further evidenced in this review, as practices documented in older studies (such as question-answer sequences) were also identified in those conducted more recently.

### Implications in the context of relevant guidelines and literature

Existing literature shows clinicians have identified health behaviour change talk (HBCT) as difficult to initiate due to its often delicate nature [7], which they are concerned may cause offence [37, 38]. This review identified three strategies clinicians used to initiate these conversations, and provided evidence on patient responses. One strategy likely to be successful is to capitalise on patient initiated HBCT. Clinicians report being more comfortable discussing HBC when the patient initiates the topic [6]. In line with this, we found strong evidence that patients are likely to be more receptive when they have initiated these discussions. Therefore, patient initiation provides good opportunities to engage in HBCT. There was no evidence presented on how doctors can best move forwards with behaviour change talk after the patient has initiated the topic. However, as the patients has raised the topic and demonstrated receptivity, one strategy could be to use collaborative health behaviour change talk, further inviting and accommodating the patient’s perspective during subsequent advice giving.

Guidelines largely offer advice for HBCT that our review suggests would be would be well-received, including goal setting [39, 40], and tailoring advice to an individual [3, 41]. However, whilst guidelines recommend these strategies they offer little support for how to implement them. The studies reviewed here showed that clinicians were using these strategies, and there was variation in how they were delivered. Having reviewed this variation we were able to identify ways that recommendations were implemented that seemed more likely to be well received, and make the following recommendations on ways to implement these guideline-recommended HBC strategies.

Guidelines advise clinicians to set goals [39, 40], and arrange appointments to review these goals at one month following a HBC discussion [3, 4]. Our review has shown that, during these review appointments, it is important to positively reinforce a patient’s efforts when reviewing their actions to change behaviours. We found that patients were held accountable for failure to meet clinical outcomes (such as weight loss), rather than on whether or not they had succeeded in changing their behaviours. This resulted in patient resistance displays. An alternative would be for clinicians to help a person see failure as learning. We saw no examples, but literature indicates this might be effective [42].

We have shown that HBC advice could be delivered as personally relevant, which is recommended by guidelines. Evidence showed that personalising by inviting and accommodating the patient’s perspective, collaborating with patients, and presenting decisions as the patient’s choice was likely to be well received. Alternatively, we found HBCT could also be framed as advice for ‘patients in general’. This was unlikely to produce resistance from patients, but the authors also hypothesise this may not motivate changes to health behaviours.

Guidelines advise associating health behaviours with current or potential health conditions [3, 39, 40, 43] and studies of clinicians’ views of HBCT show that this strategy is reported to be used frequently in practice to initiate discussions [44]. However, we found mixed evidence of effectiveness. Our results here showed that linking health behaviours and health to initiate conversations may generate resistance displays. This is a potentially risky strategy to initiate HBC and may be best avoided or used cautiously. However, linking to a salient concern later in the discussion could be a helpful way to address resistance.

Fear of causing offence when carrying out HBCT is a key concern reported by clinicians in existing studies [6, 7, 37]. Although guidelines mention the delicacy of these discussions they provide little support on how to deal with resistance if it does arise [4, 39, 45]. Most studies in this review also oriented to the delicacy of HBCT and its potential for generating resistance displays from patients, but additionally offered ways to manage resistance. This included changing the topic to talk about less delicate matters.

Clinical guidelines often recommend closing HBCT by referring patients to programmes that support behaviour change and giving practical advice on how to change [3, 39, 43]. We did not see evidence of this, and identified a clear paucity of literature on closing HBCT. The limited evidence available showed that closing by providing non-specific advice does not generate resistance. However, this may be unlikely to motivate behaviour change.

Much literature on talking about health behaviour change has focussed on motivational interviewing (MI). This process is collaborative and person-centred and aims to motivate patients to change their behaviours. Although no studies in this review used MI, a number of our results highlight aspects of the MI approach. MI, for example aims to avoid direct confrontation when discussing behaviour change [46]. In line with results from MI studies [47, 48] our results which showed that dealing with resistance through direct persuasion escalated resistance displays. Secondly, a fundamental aspect of MI is to take a client-centred approach [46]. Our results align with this aspect of MI theory identifying that collaborating with patients was likely to be a successful way to facilitate engagement in behaviour change talk. This paper has highlighted that aspects of health behaviour change used in MI, may also be successful when clinicians are not using an MI approach.

In general, these results complement current guidelines providing further detail on how they can be successfully implemented in practice. A key exception is ‘linking’ health behaviours and health, which is currently a recommended strategy for clinicians to use, but one which may generate resistance if used to initiate discussions.

More research is needed on how to deliver HBCT in ways which can motivate patient uptake of HBCT, but avoid generating resistance. Clinical trials of brief interventions have shown that they are effective in motivating behaviour change and that interventions are well received [49, 50]. Further research could explore conversational strategies used by clinicians in these studies which motivate action on health behaviours. Existing conversation analytic research has shown that patient responses to HBCT in-consultation are associated with subsequent action [51], so it is possible that the responses shown here to generate uptake displays may also be associated with behaviour change.

## Conclusions

Clinical guidelines encourage healthcare professionals to engage in HBCT with their patients [3, 4, 40, 43]. However, the difficulties in engaging in these often-sensitive discussions are well documented [7, 52, 53]. This review has shown that there are different ways that these conversations can be initiated and carried out, which can mitigate their sensitivity such as delivering HBCT in a general, non-personal way. We found evidence that is mostly consistent with current guidelines, providing further detail on how they can be successfully implemented in practice. However, one practice recommended by clinical guidelines; initiating discussions by associating a patient’s health concerns and their health behaviours, is potentially risky and can prompt patients to resist HBC. On the other hand, building conversations collaboratively by inviting patient’s views, and tailoring discussions through question-answer sequences may be well received and facilitate patient receptivity to changing their health behaviours. Clinicians can adapt themselves to the delicacy of giving advice that may have not been asked for by depersonalising it and talking ‘in theory’ or about people in general. Future work might build on the categorisation of HBCT we have developed and examine associations between behaviour change talk, and patient action on their health behaviours. Meanwhile the evidence presented here should reassure clinicians that there are several ways of starting and pursuing HBCT that patients respond to well and they need not feel so anxious when they use these approaches.

## Additional file

Additional file 1: Detailed search strategy. (DOCX 32 kb)

## Data Availability

Data were aggregated from published work where people had given consent for quotes to be shared.

## Abbreviations

HBC: Health behaviour change
HBCT: Health behaviour change talk

## References

[1] Talking with Patients about Weight Loss: Tips for Primary Care Providers [https://www.niddk.nih.gov/health-information/weight-management/talking-adult-patients-tips-primary-care-clinicians]. Accessed 7 Jan 2017.

[2] Panel TUaDG (2008). Clinical interventions for tobacco use and dependence, treating tobacco use and dependence: 2008 update.

[3] NICE Clinical Knowledge Summaries (CKS). Smoking cessation - summary: National Institute for Health and Care Excellence; 2012. https://cks.nice.org.uk/smoking-cessation. Accessed 2 Apr 2016.

[4] Obesity prevention Clinical Guideline [CG43] [https://www.nice.org.uk/guidance/cg43]. Accessed 2 Apr 2016.

[5] Clutterbuck D J, Flowers P, Barber T, Wilson H, Nelson M, Hedge B, Kapp S, Fakoya A, Sullivan A K (2012). UK national guideline on safer sex advice. International Journal of STD & AIDS.

[6] Alexander SC, Ostbye T, Pollak KI, Gradison M, Bastian LA, Brouwer RJ (2007). Physicians’ beliefs about discussing obesity: results from focus groups. Am J Health Promot.

[7] Michie S (2007). Talking to primary care patients about weight: a study of GPs and practice nurses in the UK. Psychol Health Med.

[8] Malterud K, Ulriksen K (2011). Obesity, stigma, and responsibility in health care: a synthesis of qualitative studies. Int J Qual Stud Health Well Being.

[9] Merrill E, Grassley J (2008). Women’s stories of their experiences as overweight patients. J Adv Nurs.

[10] Epstein L, Ogden J (2005). A qualitative study of GPs’ views of treating obesity. BJGP.

[11] McCarthy DM, Waite KR, Curtis LM, Engel KG, Baker DW, Wolf MS (2012). What did the doctor say? Health literacy and recall of medical instructions. Med Care.

[12] Schegloff EA (2007). Sequence organization in interaction : a primer in conversation analysis I.

[13] Parry R, Land V, Seymour J (2014). How to communicate with patients about future illness progression and end of life: a systematic review. BMJ Support Palliat Care.

[14] ten Have P (2007). Doing conversation analysis: a practical guide.

[15] Traynor M (2006). Discourse analysis: theoretical and historical overview and review of papers in the Journal of Advanced Nursing 1996–2004. J Adv Nurs.

[16] Niemants N, Stokoe E (2017). Using the conversation analytic role-play method in healthcare interpreter training. Teaching dialogue interpreting.

[17] Let’s Talk About Weight: A step-by-step guide to brief interventions with adults for health and care professionals [https://www.gov.uk/government/publications/adult-weight-management-a-guide-to-brief-interventions]. Accessed 22 June 2017.

[18] Let’s Talk About Weight:A step-by-step guide to conversations about weight management with children and families for health and care professionals [https://www.gov.uk/government/uploads/system/uploads/attachment_data/file/649095/child_weight_management_lets_talk_about_weight.pdf]. Accessed 19 Oct 2017.

[19] Kent A (2012). Compliance, resistance and incipient compliance when responding to directives. Discourse Stud.

[20] Muntigl P (2013). Resistance in couples counselling: sequences of talk that disrupt progressivity and promote disaffiliation. J Pragmat.

[21] Parry RH, Land V (2013). Systematically reviewing and synthesizing evidence from conversation analytic and related discursive research to inform healthcare communication practice and policy: an illustrated guide. BMC Med Res Methodol.

[22] Thomas J, Harden A (2008). Methods for the thematic synthesis of qualitative research in systematic reviews. BMC Med Res Methodol.

[23] Ziebland S, McPherson A (2006). Making sense of qualitative data analysis: an introduction with illustrations from DIPEx (personal experiences of health and illness). Med Educ.

[24] Barnett-Page E, Thomas J (2009). Methods for the synthesis of qualitative research: a critical review. BMC Med Res Methodol.

[25] Cohen DJ, Clark EC, Lawson PJ, Casucci BA, Flocke SA (2011). Identifying teachable moments for health behavior counseling in primary care. Patient Educ Couns.

[26] Collins S, Drew P, Watt I, Entwistle V (2005). ‘Unilateral’ and ‘bilateral’ practitioner approaches in decision-making about treatment. Soc Sci Med.

[27] Freeman SH (1987). Health promotion talk in family practice encounters. Soc Sci Med.

[28] Kinnell AM, Maynard DW (1996). The delivery and receipt of safer sex advice in pretest counseling sessions for HIV and Aids. J Contemp Ethnogr.

[29] Pilnick A, Coleman T (2003). ‘I’ll give up smoking when you get me better’: patients’ resistance to attempts to problematise smoking in general practice (GP) consultations. Soc Sci Med.

[30] Pilnick A, Coleman T (2010). ‘Do your best for me’: the difficulties of finding a clinically effective endpoint in smoking cessation consultations in primary care. Health.

[31] Silverman D, Bor R, Miller R, Goldman E, Aggleton P, Davies P, Hart G (1992). ‘Obviously the advice is then to keep to safer sex’: advice-giving and advice reception in AIDS counselling. AIDS: rights, risk and reason.

[32] Silverman D, Perakyla A, Bor R (1992). Discussing safer sex in HIV counselling: assessing three communication formats. AIDS Care.

[33] Tapsell L (1997). Client-centred practice: an interactional case study of dietary counselling. Health.

[34] Thille P, Ward N, Russell G (2014). Self-management support in primary care: enactments, disruptions, and conversational consequences. Soc Sci Med (1982).

[35] Pomerantz A (1990). Conversation analytic claims. Commun Monogr.

[36] Maynard D (2013). Everyone and no one to turn to: intellectual roots and contexts for conversation analysis.

[37] Blackburn M, Stathi A, Keogh E, Eccleston C (2015). Raising the topic of weight in general practice: perspectives of GPs and primary care nurses. BMJ Open.

[38] Miller ER, Ramsey IJ, Tran LT, Tsourtos G, Baratiny G, Manocha R, Olver IN (2016). How Australian general practitioners engage in discussions about alcohol with their patients: a cross-sectional study. BMJ Open.

[39] Alcohol-use disorders: prevention [https://www.nice.org.uk/guidance/ph24]. Accessed 2 Apr 2016.

[40] Obesity: guidance on the prevention, identification, assessment and management of overweight and obesity in adults and children. [https://www.nice.org.uk/guidance/cg189]. Accessed 2 Apr 2016.

[41] Top Ten Tips Raising the Topic of Weight [https://www.rcgp.org.uk/-/media/Files/CIRC/Clinical-News/Top-Ten-Tips-Leaflet-2013.ashx?la=en]. Accessed 19 Oct 2017.

[42] Kangovi S, Asch DA (2018). Behavioral phenotyping in health promotion: Embracing or avoiding failure. JAMA.

[43] Weight management: lifestyle services for overweight or obese adults [https://www.nice.org.uk/guidance/ph53/resources/weight-management-lifestyle-services-for-overweight-or-obese-adults-pdf-1996416726469]. Accessed 2 Apr 2016.

[44] Claridge R, Gray L, Stubbe M, Macdonald L, Tester R, Dowell AC (2014). General practitioner opinion of weight management interventions in New Zealand. J Prim Health Care.

[45] NICE (2018). Stop smoking interventions and services.

[46] Rollnick S, Miller WR (1995). What is motivational interviewing?. Behav Cogn Psychother.

[47] Miller WR, Benefield RG, Tonigan JS. Enhancing motivation for change in problem drinking: a controlled comparison of two therapist styles. J Consult Clin Psychol. 1993;61(3):455-61. 10.1037//0022-006x.61.3.455 8326047

[48] Miller WR, Rollnick S (1991). Motivational Interviewing: preparing people to change addictive behavior.

[49] Aveyard P, Begh R, Parsons A, West R. Brief opportunistic smoking cessation interventions: a systematic review and meta-analysis to compare advice to quit and offer of assistance. (1360-0443 (Electronic)). 10.1111/j.1360-0443.2011.03770.x 22175545

[50] Aveyard P, Lewis A, Tearne S, Hood K, Christian-Brown A, Adab P, Begh R, Jolly K, Daley A, Farley A (2016). Screening and brief intervention for obesity in primary care: a parallel, two-arm, randomised trial. Lancet.

[51] Albury CVA, Stokoe E, Ziebland S, Webb H, Aveyard P (2018). GP-delivered brief weight loss interventions: a cohort study of patient responses and subsequent actions, using conversation analysis in UK primary care. Br J Gen Pract.

[52] Gott M, Galena E, Hinchliff S, Elford H (2004). “Opening a can of worms”: GP and practice nurse barriers to talking about sexual health in primary care. Fam Pract.

[53] Vogt F, Hall S, Marteau TM (2005). General practitioners’ and family physicians’ negative beliefs and attitudes towards discussing smoking cessation with patients: a systematic review. Addiction.

