# Supplementary material for: Communication practices for delivering health behaviour change conversations in primary care: a systematic review and thematic synthesis
Source: BMC Fam Pract. 2019 Aug 3;20:111. doi: 10.1186/s12875-019-0992-x (PMC6679536; doi:10.1186/s12875-019-0992-x)
Supplement: Supplementary file 1 — Detailed search strategy. (DOCX 32 kb) [file 12875_2019_992_MOESM1_ESM.docx]

Data Sources:

We searched the following databases: MEDLINE(OvidSP)[1946-present]; Embase(OvidSP)[1974-present]; Web of Science Core Collection(Thomson Reuters)[1945-present]; AMED(OvidSP)[1985-present]; CINAHL(EBSCOHost)[1982-present]; PsycINFO(OvidSP)[1967-present]; Scopus; Sociological Abstracts(CSA)[1952-present], and restrictions were applied to specify human subjects and English language. We searched peer-reviewed journals and published book chapters from 1945 onwards. The following resources were additionally searched: bibliographies of included full-texts; specialist online discussion lists; and review team knowledge and contacts. All searches were conducted from January to March 2016. Searches were updated in March 2018. This comprehensive research strategy was designed with advice from an information specialist (NR). The full search strategy is available in Additional file 1.

# Additional File 1:

The first search strategy was designed to identify literature which focusses on a specific health behaviours (such as weight loss, or smoking cessation), and the second was designed to identify literature which may focus on a behaviourally-related action (such as adherence or motivation), rather than specific behaviour.

Search Strategy 1: Medline Ovid

| # ▲ | | Searches |
| --- | --- | --- |
| 1 | (medic* or treatment* or care* or healthcare* or health* or patient* or doctor* or clinic* or physician* or primary care* or consult* or general practi* or family practi*).mp. | |
| 2 | ("conversation analysis" or "conversational analysis" or "conversation analyses" or "conversational analyses" or "conversation analytic" or "conversational analytic" or "discourse analysis" or "discourse analytic" or "discourse psychology" or "sequential-analysis" or "conversation-analysis conversational-analysis" or "conversation-analyses" or "conversational-analyses" or "conversation-analytic" or "conversational-analytic" or "talk in interaction" or "talk-in-interaction" or linguistic*).mp. | |
| 3 | ("weight loss" or smok* or alcohol* or lifestyle* or intervention* or diet* or exercise* or "physical activity" or activ* or "blood pressure" or diabetes or "tobacco use" or "physical inactivity" or "body weight" or cholesterol or "stress management" or "sexual health" or "organ donation" or "life support" or vaccinat* or weigh*).mp. | |
| 4 | (audio or video).mp. | |
| 5 | 1 and 2 and 3 and 4 | |

Search Strategy 2: Medline Ovid

| # ▲ | Searches |
| --- | --- |
| 1 | (medic* or treatment* or care* or healthcare* or health* or patient* or doctor* or clinic* or physician* or primary care* or consult* or general practi* or family practi*).mp. |
| 2 | ("conversation analysis" or "conversational analysis" or "conversation analyses" or "conversational analyses" or "conversation analytic" or "conversational analytic" or "discourse analysis" or "discourse analytic" or "discourse psychology" or "sequential-analysis" or "conversation-analysis conversational-analysis" or "conversation-analyses" or "conversational-analyses" or "conversation-analytic" or "conversational-analytic" or "talk in interaction" or "talk-in-interaction" or linguistic*).mp. |
| 3 | (motivat* or coerc* or recommend* or interven* or resist* or incentiv* or encourage* or accept* or "decision making" or ahere* or cooperat* or empower* or instigat* or initiat* or "behaviour change" or "behavior change" or "behavioural change" or "behavioral change" or "behavioural changes" or "behavioral changes" or persuad* or choice* or uptake* or nudge* or convinc* or concord* or communicat* or agen* or pressur* or negotiat* or comply or compliance or enable* or co-operat* or facilitat* or barrier* or disincentive* or refusal).mp. |
| 4 | Physician-Patient Relations/ or Health Communication/ or Patient Compliance/ or Medication Adherence/ or Patient Satisfaction/ or Treatment Refusal/ or Negotiating/ or health behaviour/ or decision making/ or motivation/ or health behavior/ |
| 5 | 3 or 4 |
| 6 | (audio or video).mp. |
| 7 | 1 and 2 and 5 and 6 |
